# Supplementary material for: WordCommentsAnalyzer: A windows software tool for qualitative research
Source: F1000Res. 2018 Sep 4;7:536. Originally published 2018 May 3. [Version 2] doi: 10.12688/f1000research.14819.2 (PMC6137411; doi:10.12688/f1000research.14819.2)
Supplement: Supplementary file 3 [file f1000research-7-17614-s0002.tgz › b2a79496-4ada-47c4-9682-a99ce867836d.zip!/Eight Iranian Health Journals/Journal of Health System Research _47.docx]

© Journal of Health System Research . This work is licensed under CC BY-NC 4.0 (https://creativecommons.org/licenses/by-nc/4.0/).

Volume & Issue: دوره 6، شماره 2؛ 1389

Title: توصیف عادات غذایی دختران جوان اصفهانی: وضعیت دریافت چای، غذاهای‌آماده،چربی‌ها

http://hsr.mui.ac.ir/index.php/jhsr/article/view/122

مقدمه: مصرف زیاد چای، غذاهای‌ آماده با بیماری‌های مزمن نظیر اضافه وزن و پرفشاری‌خون در‌ ارتباط می‌باشد. با توجه به اهمیت پیشگیری از بیماری‌های مزمن در سنین جوانی هدف از این مطالعه توصیف عادات غذایی در دختران جوان اصفهانی می‌باشد.  روش‌ها: در این مطالعه مقطعی 110 دختر جوان 18تا 28 ساله دانشگاه علوم پزشکی اصفهان به روش نمونه‌گیری خوشه‌ای سیستماتیک و تصادفی انتخاب شدند. جهت ارزیابی عادات غذایی از یک پرسش‌نامه اعتبارسنجی شده، استفاده گردید. شاخص‌های آنتروپومتریک و فشارخون با استفاده از روش‌‌های استاندارد ارزیابی شد. نمایه توده بدنی بالاتر از 25کیلوگرم بر مترمربع به عنوان اضافه وزن و بیشتر از 30 کیلوگرم بر مترمربع به‌ عنوان چاق تلقی گردید. دور کمر بیشتر از 85 سانتی‌متر به‌ عنوان چاقی شکمی تعریف شد. یافته‌ها: 51 درصد دختران جوان کمتر از 3 لیوان در روز چای و 40 درصد آنها 3 تا 5 لیوان و 9 درصد بیشتر از 5 لیوان چای می‌نوشیدند. تنها 2 درصد  روزانه از قهوه استفاده می‌کردند. 50 درصد افراد از غذاهای آماده استفاده نمی‌کردند و 5/44 درصد آنها 2بار در هفته از غذاهای آماده و 5/5 درصد آنها بیشتر از 2 بار در هفته از غذاهای آماده استفاده می‌‌‌‌کردند. 5/44 درصد از جوانان از غذاهای چرب و 5/54 درصد از غذاهای کم‌چرب استفاده می‌‌‌کردند. روغن مصرفی 5/54 درصد روغن مایع، 4/36 درصد روغن مخصوص سرخ‌کردنی و 3/7 درصد جامد و 8/1 درصد از روغن حیوانی بود. نتیجه‌گیری: استفاده از غذاهای چرب و مصرف غذاهای آماده از عادات غذایی نادرست در دختران جوان بود. بر این اساس، ضرورت اصلاح عادات غذایی و روش‌های پخت در قشر جوان کشورمان احساس می‌شود.

Title: ارزش‌یابی طرح مشارکت مادران در روند رشد و مراحل تکامل کودکان بر اساس مدل CIPP

http://hsr.mui.ac.ir/index.php/jhsr/article/view/31

مقدمه: بررسي كشوري ارزيابي رشد كودكان نشان داده است كه درصد زيادي از كودكان كشور دچار افت رشد هستند. علاوه بر آن در گذشته طرح‌های زیادی در زمینه رشد و تغذیه اطفال انجام شده است ولی می‌توان گفت که اکثریت آن‌ها بدون ارزش‌یابی و بررسی نتیجه رها گردیده است این مطالعه با هدف ارزش‌یابی طرح مشارکت مادران (جلب مشارکت مادران در بهبود رشد و تغذیه اطفال بر اساس مدل Context, Input, Process Product (CIPP)) پس از گذشت دو سال انجام شده است.  روش‌ها: این مطالعه از نوع مطالعات توصیفی و در قالب مطالعات ارزش‌یابی است که در آن 90 زوج مادر و کودک در قالب دو گروه مشارکت‌کننده در طرح و یک گروه شاهد (همسان شده با گروه شرکت کننده در طرح) جهت بررسی نتایج مورد مطالعه قرار گرفتند. ابزار گردآوری اطلاعات در مطالعه حاضر فرم پایش رشد کودک و پرسش‌نامه مورد استفاده در طرح مشارکت مادران بوده است و اطلاعات از طریق مصاحبه سازمان یافته، جمع‌آوری و با استفاده از نرم افزار SPSS15 و آزمون‌های آماری مناسب مورد تجزیه و تحلیل قرار گرفته است. یافته‌ها: نتایج مطالعه اختلاف معنی‌دار بین دو گروه مورد بررسی در میانگین نمره اعتماد به نفس مادران (001/0> P)، عملکرد مادران در آموزش دادن به سایرین (006/0= P)، مدت زمان مطالعه (004/0=P ) و فراوانی مادران، شرکت در برنامه‌های آموزشی (002/0= P)، آشنا به کارت پایش رشد (03/0= P)، دارای عملکرد صحیح رسم منحنی رشد (001/0> P)، آگاه از انواع منحنی‌های رشد (001/0= P) و مادران آگاه در زمینه اهداف پایش رشد (001/0> P) نشان می‌دهد. نتیجه‌گیری: نتایج نشان از ماندگاری اثرات آموزش‌های ارایه شده در طرح مشارکت مادران و بهبود آگاهی و عملکرد مادران در خصوص رشد و تکامل کودکان پس از گذشت دو سال دارد که این امر کارآیی مدل مورد استفاده (CIPP) در برنامه آموزشی نشان می‌دهد.

Title: ارزیابی فعالیت جسمانی دانشجویان پزشکی و بهداشت دانشگاه علوم پزشکی اصفهان 1387

http://hsr.mui.ac.ir/index.php/jhsr/article/view/59

مقدمه: کاهش فعالیت‌های جسمانی یکی از معضلات شهرنشینی و توسعه صنعتی است که در ایجاد بیمار‌ي‌ها، ناتوانی‌ها و مرگ و میر تأثیر بسزایی دارد. در کشورهای مختلف بین60 تا 85 درصد از بزرگسالان تحرک کافی برای بهبود سلامتی ندارند و زندگی بی‌تحرک باعث افزایش تمام علل مرگ و میر می‌شود. بنابراین کم‌تحرکی در جوانان اهمیت زیادی دارد، که در این مطالعه به منظور بهبود شیوه زندگی این قشر، میزان فعالیت بدنی آنان مورد ارزیابی قرار گرفت.  روش‌ها: این مطالعه به صورت مقطعی بر روی 399 نفر از دانشجویان دانشکده‌های پزشکی و بهداشت دانشگاه علوم پزشکی اصفهان صورت گرفت. به منظور ارزیابی فعالیت جسمانی دانشجویان از پرسش‌نامه استانداردی استفاده شد که فعالیت جسمانی را در چهار حیطه شغل، جابجایی، کار در منزل و اوقات فراغت مورد پرسش قرار می‌داد. داده‌ها پس از جمع‌آوری با روش‌هاي آمار تحليلي تجزیه و تحلیل گردید. یافته‌ها: نتایج ارزیابی فعالیت 399 نفر دانشجو (شامل4/66 درصد دختر و 6/33 درصد پسر) نشان دادند که 6/48 درصد از دانشجویان از نظر فعالیت جسمانی متوسط، فعال و 4/51 درصد غیرفعال بودند. همچنین از نظر فعالیت جسمانی شدید 6/32 درصد فعال و 4/76 درصد غیرفعال بودند. میانگین و انحراف معيار تعداد ساعت فعالیت متوسط در روز 19/0 ± 96/1 ساعت است. در این مطالعه رابطه سن، جنس، رشته تحصیلی و طبقه اجتماعی دانشجویان نیز با میزان فعالیت بدنی آن‌ها در حیطه‌های مختلف بررسی شد که از نظر جنس و رشته تحصیلی رابطه معنی‌داری به دست آمد. نتیجه‌گیری: با توجه به اهمیت فعالیت جسمانی در زندگی جوانان، نتایج مطالعه نشان می دهند که فعالیت جسمانی در جامعه مورد بررسی کافی نیست و لازم است با برنامه‌ریزی صحیح و مستمر، آموزش‌های لازم در خصوص تغییر شیوه زندگی دانشجویان به ویژه در زمینه جابجایی و تحرک در اوقات فراغت به آن‌ها داده شود.

Title: مقايسه وضعيت تغذيه‌اي نوزادان كم‌وزن و طبيعي در رابطه با رشد آن‌ها در بيمارستان‌هاي وابسته به دانشگاه علوم پزشکی اصفهان

http://hsr.mui.ac.ir/index.php/jhsr/article/view/62

مقدمه: هرساله 21 ميليون نوزاد كم‌وزن و نارس در جهان متولد مي‌شوند. مطالعه‌ها نشان مي‌دهند كه نوزادان نارس و كم‌وزن در مقايسه با نوزادان طبيعي از شانس زنده ماندن كمتري برخوردارند.‌ اين پژوهش به منظور مقايسه وضعيت رشد بازيافته نوزادان كم‌وزن و نارس با نوزادان طبيعي انجام گرديد.  روش‌ها: پرسش‌نامه‌اي شامل متغيرهاي تعيين شده براي مراحل مختلف پژوهش تهيه گرديد و در هنگام تولد و مقاطع سه ماهگي، شش ماهگي به تعداد نمونه‌هاي برآورد شده كه شامل 214 نوزاد كم‌وزن، 460 نوزاد بسيار كم‌وزن و 38 نوزاد به شدت كم‌وزن بودند، تكميل گرديد. نمونه‌ها از بين متولدين بيمارستان‌هاي الزهرا و شهيد بهشتي انتخاب شدند. یافته‌ها: 92 درصد از نوزادان طبيعي به وسيله شير مادر، 5 درصد از آن‌ها به روش گاواژ و 2 درصد از طريق بطري تغذيه شدند. متوسط وزن تولد نوزادن طبيعي، كم‌وزن، بسيار كم‌وزن و به شدت كم‌وزن به ترتيب برابر 3114، 2058، 1348 و 953 گرم به دست آمد. نتیجه‌گیری: درصد نوزاداني كه از گاواژ و شير مادر استفاده كردند با افزايش كم‌وزني نوزادان، افزايش مي‌يابد. مقايسه ميانگين وزن نوزادان طبيعي، كم‌وزن، بسيار كم‌وزن و به شدت كم‌وزن در هنگام تولد، يك ماهگي، سه ماهگي و شش ماهگي نشان می‌دهد كه نوزادان طبيعي و كم‌وزن تا سن شش ماهگي ‌اين تفاوت وزن را حفظ كردند ولی در بين نوزادان بسيار كم‌وزن و به شدت كم‌وزن شش ماهه ‌اين تفاوت مشاهده نمي‌شود، در حالي كه تفاوت وزن آن‌ها با نوزادان طبيعي همچنان باقي مانده است.

Title: بررسي ويژگي‌هاي تن‌سنجي دانش‌آموزان دبستاني اصفهان در راستاي طراحي اندازه ميز و نيمكت كلاس

http://hsr.mui.ac.ir/index.php/jhsr/article/view/63

مقدمه: طراحي صحيح وسايل و تجهيزات مدارس، متناسب با خصوصيات تن‌سنجي استفاده كنندگان، جهت ايجاد عادت صحيح نشستن و گرفتن وضعيت بدني مناسب در استفاده كنندگان از اين وسايل، بسيار مهم می‌باشد. ميز و نيمكت‌هايي كه در حال حاضر در مدارس ايران مورد استفاده قرار مي‌گيرند، متناسب با ابعاد فيزيكي دانش‌آموزان محلي طراحي نشدند. هدف اين پژوهش ارایه توصيه‌هايي در زمينه طراحي صحيح ميز و نيمكت‌هاي مورد استفاده در مؤسسات آموزشي متناسب با ابعاد فيزيكي دانش‌آموزان می‌باشد.  روش‌ها: در اين تحقيق تعداد 300 دانش‌آموز دختر و پسر ناحيه 3 شهرستان اصفهان به صورت تصادفي منظم انتخاب شدند و اطلاعات لازم توسط مصاحبه و مشاهده و با استفاده از وسايل اندازه‌گيري آنتروپومتريكي در پرسش‌نامه ثبت گرديد. یافته‌ها: اطلاعات آنتروپومتريكي براي300 دانش‌آموز دختر و پسر، از 17 پارامتر آنتروپومتريكي با توجه به چك ليستي كه براي هر نفر تكميل شده بود به دست آمد و با استفاده از نرم‌افزار SPSS مورد تجزيه و تحليل قرار گرفت. آزمون t-test بين پارامترهاي دختران و پسران نشان می‌دهد كه تنها در پارامترهاي عمق سينه، ارتفاع ركبي، طول كفل، زانو، طول كفل- ركبي، عمق ران و حد دسترسي جلو بازو اختلاف معني‌داری وجود دارد. نتیجه‌گیری: بر اساس نتايج، مشخص می‌شود كه ارتفاع ميز بر اساس حاصل جمع صدك 95 ارتفاع تكيه‌گاه آرنج و ارتفاع سطح نشستن‌گاه جامعه مورد مطالعه به دست مي‌آيد. با اندازه‌هايي كه در ميزهاي فعلي وجود دارد، دانش‌آموزان مجبور مي‌شوند كه روي نيمكت خود ننشيند و بايستند، كه اين ايستادن بدون حركت در ساعت‌هاي متمادي موجب واريس سياهرگي در پا و ساق پا مي‌شود.

Title: رابطه‌ی ویژگی‌های زیستی، شناختی روانی و اجتماعی- فرهنگی والدین با کم وزنی هنگام تولد

http://hsr.mui.ac.ir/index.php/jhsr/article/view/86

مقدمه: کم وزنی هنگام تولد، یکی از شاخص‌های مهم بهداشتی هر کشور است و شناخت عوامل آن ضروری می‌باشد. پژوهش حاضر، رابطه‌ی بین ویژگی‌های زیستی، شناختی، روانی و اجتماعی والدین با کم وزنی هنگام تولد در استان اصفهان بود.  روش‌ها: در این پژوهش توصیفی، 910 نفر (از بین مادران تازه زایمان کرده) تمامی نوزادان متولد شده در بیمارستان‌ها و زایشگاه‌های دولتی وخصوصی استان اصفهان به روش نمونه‌گیری طبقه‌ای و خوشه‌ای در سال 1388 انتخاب شدند. اطلاعات با پرسش‌نامه‌ای محقق و آزمون رضایت زناشویی اینریچ جمع‌آوری شد. داده‌ها با نرم افزار SPSS تجزیه وتحلیل گردید. یافته‌ها: شیوع نوزادان کم وزن 86 نوزاد ( 5/9 درصد)، بارداری‌های ناخواسته 35 مورد ( 7/38 درصد)، نارضایتی زناشویی مادران 294 نفر ( 29 درصد) بوده 142 نفر (6/15 درصد ) بارداری‌ها زیر 20 سال، 200 نفر (22 درصد) بالای 35 سال و 354 نفر (9/38 درصد) در معرض دود سیگار، 300 نفر (33 درصد) فشار خون بالا، 242 نفر ( 7/26 درصد) سابقه‌ی سقط جنین و 290 نفر ( 9/31 درصد) خون‌ریزی داشتند 95 نفر ( 4/10 درصد) نوزادان داراي خواهر يا برادر استثنايي بودند .210 تفر ( 1/23 درصد) مادران شاغل، 180 نفر ( 8/19 درصد) تولد دوقلویی و 192 نفر ( 1/21 درصد) والدین نسبت خویشاوندی نزدیک داشتند میانگین افزایش وزن دوران بارداری 9 کیلوگرم، 270 نفر ( 7/29 درصد) زایمان‌ها سزارین بود این عوامل خطرزا، می‌تواند احتمال کم‌وزنی هنگام تولد را به طور معناداری افزایش دهد (05/0 = a). نتیجه‌گیری: ویژگی‌های زیستی، روانی و اجتماعی والدین بر کم وزنی هنگام تولد تاثیر داشته و در پیشگیری ازتولد نوزادان کم وزن کاربرد دارد.

Title: بررسی عملکرد فرایند ازن‌زنی کاتالیزوری با پامیس درحذف اسید هیومیک از محلول‌های آبی

http://hsr.mui.ac.ir/index.php/jhsr/article/view/113

مقدمه: در سال‌های اخیر استفاده از گندزداهای مختلف و کنترل ترکیبات آلی طبیعی، دو روشی هستند که عموماً در تصفیه آب برای کاهش تشکیل محصولات جانبی کلرزنی به کار برده می‌شوند. ازن‌زنی کاتالیزوری، یک روش جدید برای افزایش راندمان فرایند ازن‌زنی متداول می‌باشد. این مطالعه با هدف بررسی امکان‌سنجی استفاده از پامیس به عنوان کاتالیزور در فرایند ازن‌زنی جهت حذف اسید هیومیک از محلول‌های آبی، صورت گرفت. روش‌ها: این مطالعه یک مطالعه تجربی می‌باشد که در آزمایشگاه شیمی آب و فاضلاب دانشگاه علوم پزشکی همدان انجام شد. جهت تعیین ساختار ملکولی و ترکیب شیمیایی پامیس، از تکنیک پراکنش پرتو ایکس (XRF) و XRD استفاده شد. آزمایش‌های ازن‌زنی کاتالیزوری در راکتور نیمه منقطع انجام شد و غلظت ازن به روش یدومتری تعیین گردید. تغییرات غلظت اسيد هيوميك در نمونه‌ها با استفاده از اسپکتروفتومتر در طول موج 254 نانومتر تعیین شد. تأثیر پارامترهای مختلف از قبیلpH ، زمان تماس، دوز پامیس و ترت بوتیل الکل در فرایند حذف اسید هیومیک بررسی شد و نتایج به دست آمده با نرم‌افزار اکسل مورد تجزیه و تحلیل قرار گرفت. یافته ها: استفاده از پامیس به عنوان کاتالیزور، سرعت تجزیه اسید هیومیک را تا 8 برابر افزایش می‌دهد و با افزایش pH از 4 تا 12 و دوز کاتالیزور از 25/0 تا 10 گرم، راندمان حذف افزایش می‌یابد. فرایند کاتالیزوری در مقایسه با فرایند ازن‌زنی متداول، اسید هیومیک را در زمان کمتری (10 دقیقه) با راندمان قابل توجهی (90 درصد) حذف می‌کند. علاوه بر آن، نتایج به دست آمده نشان می‌دهند تأثیر عوامل مداخله‌کننده درفرایند ازن‌زنی کاتالیزوری در مقایسه با فرایند ازن‌زنی متداول قابل توجه نیست. نتیجه‌گیری: به دليل هزینه کم، دسترسي آسان به مواد اوليه و عملکرد قابل توجه پامیس به عنوان کاتالیزور، این سنگ می‌تواند به عنوان کاتالیزور در فرایند ازن‌زنی متداول برای حذف آلاینده‌های آلی به کار برده شود.

Title: آگاهی، نگرش و عملکرد استادان در خصوص مهارت‌های ارتباطی مؤثر در آموزش

http://hsr.mui.ac.ir/index.php/jhsr/article/view/127

مقدمه: ارتباطات یک بخش مهم از فرآیند آموزشی را تشکیل می‌دهد که هدف آن انتقال یا تبادل ایده‌ها و افکار می‌باشد و تأمین آن منوط به وجود مهارت‌های ارتباطی در بین اساتید دانشگاه می‌باشد. هدف از این مطالعه بررسی آگاهی، نگرش و عملکرد اساتید دانشکده بهداشت دانشگاه علوم پزشکی اصفهان در خصوص مهارت‌های ارتباطی مؤثر در آموزش می‌باشد.  روش‌ها: اين پژوهش يك مطالعه توصيفي‌ –‌ تحليلي است كه اطلاعات آن از طریق پرسشنامه‌های روا و پاياي دانش (8 سوال وحداکثر 8 امتیاز)، نگرش (31 سوال وحداکثر 155 امتیاز) و چك‌ليست مشاهده‌ای عملکرد مهارت‌های ارتباطی(20 سوال وحداکثر 20 امتیاز) از کلیه اساتید شاغل به تدریس در دانشکده بهداشت دانشگاه علوم پزشکی اصفهان درنیمسال دوم تحصیلی 85-84 جمع‌آوری گردید. تجزیه و‌تحلیل داده‌ها با استفاده از نرم‌افزار SPSS و با محاسبه شاخص‌های مرکزی انجام گردید. یافته‌ها: میانگین نمره آگاهی افراد مورد مطالعه در خصوص مهارت‌های ارتباطی 1/4 از 8 امتیاز، میانگین نمره نگرش آنان در این زمینه 4/114 از 155 امتیاز و میانگین نمره عملکرد این افراد 3/16 از 20 امتیاز بود. نتیجه‌گیری: هر چند اطلاعات افراد شرکت‌کننده در این پژوهش در زمینه مهارت‌های ارتباطی كافي به نظر نمي‌رسد، اما این افراد در مورد مهارت‌های ارتباطی، نگرش مثبت و عملکرد نسبتاً قابل قبولي دارند.

Title: وضعيت تغذيه با شير مادر تا پايان دوسالگي و عوامل مؤثر بر آن از طریق سازه‌های مدل بزنف در شهر اصفهان 1388

http://hsr.mui.ac.ir/index.php/jhsr/article/view/131

مقدمه: تغذيه با شير مادر تابع شرایط اجتماعی و فرهنگی جوامع است. مدل بزنف یکی از مدل‌های جامع مطالعه رفتار به‌ خصوص در کشورهای در حال توسعه می‌باشد. مطالعه حاضر با هدف بررسي وضعيت تغذيه با شير مادر تا پايان دوسالگي و عوامل مؤثر بر آن از طریق سازه‌های مدل بزنف انجام گرفت. روش‌ها: پژوهش حاضر توصیفیـ تحلیلی ازنوع مقطعی می‌باشد. تعداد 406 مادر دارای کودک 6 الی 24 ماهه به‌ طور نمونه‌گیری خوشه‌ای چند مرحله‌ای انتخاب شدند. اطلاعات با پرسش‌نامه‌ای مشتمل بر 5 بخش براساس سازه‌های مدل بزنف به همراه اطلاعات د‌‌‌موگرافیک جمع‌آوری گردید. و از طریق نرم‌افزار آماریspss 18  و با استفاده از آزمون‌های کای اسکوئر و آنالیز واریانس تجزیه و تحلیل شد. یافته‌ها: شیوع تغذیه با شیرمادر351 نفر ) 4/86 درصد) بود. 333 نفر (82 درصد) دارای آگاهی خوب بودند. بین سطح آگاهی و رفتار شیردهی مادران ارتباط معنی‌داری وجود داشت (p<0.05). 285نفر (2/70 درصد ) دارای نگرش مطلوب و خیلی مطلوبی بودند. بین نگرش و رفتارشیردهی مادران ارتباط آماری معنی‌دار یافت نشد (p>0.05). بین هنجارهای انتزاعی همسر، مادر و مادرشوهر با رفتار شیردهی مادران ارتباط آماری معنی‌دار وجود داشت (p<0.05) ولی در مورد عوامل قادرساز این ارتباط یافت نشد (p>0.05). همچنین بین نوع تغذیه شیرخوار و متغیر‌های نوع زایمان، تعداد فرزندان و سن مادران ارتباطی مشاهده نشد (p>0.05). نتیجه‌گیری: علاوه بر وجود آگاهی و اطلاعات مادران شیرده، حمایت و تشویق همسر، مادر و مادر شوهر بعنوان هنجارهای انتزاعی در رفتار شیردهی مادران تاثیرمثبتی دارند و در برنامه‌های تغذیه با شیر مادر به نقش این عوامل توجه بیشتری باید مبذول شود. واژه‌های‌ کلیدی: تغذيه با شير مادر، شیرخوار، مدل بزنف، عوامل قادرساز، هنجارهای انتزاعی.

Title: کیفیت میکروبی آب شناگاه‌های دریای خزر در سواحل استان گلستان

http://hsr.mui.ac.ir/index.php/jhsr/article/view/38

مقدمه: شنا به دلیل اثرات مفید روی مفاصل و احساس رضایت و شادابی عمومی مردم توصیه می‌شود. شنا کردن یک راه مهم برای تمدد اعصاب و ضربان قلب گرمای تابستان می‌باشد. تخلیه فاضلاب‌های شهری و صنعتی با سطح بالایی از پاتوژن‌ها و سایر آلاینده‌های دیگر، سلامت شناگران را تحت تأثیر قرار می‌دهد. این مطالعه به منظور ارزیابی آلودگی میکروبی آب شناگاه‌های دریای خزر در سواحل استان گلستان و مقایسه آن با استاندارد وزارت بهداشت انجام گرفت.  روش‌ها: به منظور ارزیابی وضعیت میکروبی شناگاه‌های سواحل استان گلستان، تعداد 120 نمونه آب از شناگاه شهرهای ساحلی ترکمن، بندرگز و نوکنده (هر شهر 40 نمونه) در شش ماه اول 1388 برداشت و میانگین تعداد کلیفرم‌ها، کلیفرم مدفوعی، استرپتوکوک مدفوعی و سودوموناس با روش استاندارد تعیین گردید. یافته‌ها: میانگین تعداد كلیفرم، كلیفرم مدفوعی، استرپتوكوك مدفوعی و سودوموناس در سواحل استان گلستان به ترتیب برابر 366، 249، 73، 93 و 7 در هر 100 میلی‌لیتر نمونه بود. همچنین میانگین تعداد كلیفرم، كلیفرم مدفوعی، استرپتوكوك مدفوعی و سودوموناس در شناگاه ترکمن به ترتیب برابر 505، 374، 110 و 9 در هر 100 میلی‌لیتر نمونه، در  شناگاه بندرگز به ترتیب برابر 369، 233، 28 و 7 در هر 100 میلی‌لیتر نمونه و در شناگاه نوکنده به ترتیب برابر 224، 174، 57 و 6 در هر 100 میلی‌لیتر نمونه به دست آمد. نتیجه‌گیری: این مطالعه نشان داد که میزان آلودگی از نظر میکروارگانیسم‌های شاخص در کلیه شناگاه‌های استان در ماه مرداد و اردیبهشت، دارای بالاترین و کمترین مقدار بود. از نظر کلیفرم کل و استرپتوکوک مدفوعی، فقط شناگاه ترکمن از استاندارد وزارت بهداشت بالاتر بود، ولی از نظر کلیفرم مدفوعی و سودوموناس کلیه مناطق شنای استان گلستان از استاندارد وزارت بهداشت بالاتر بود. بنابراین سواحل استان گلستان از نظر میکروب‌های شاخص دارای آلودگی می‌باشند که می‌‌تواند برای سلامتی شناگران خطرناک باشد.

Title: اندازه‌گیری و شبیه‌سازی اکسیژن محلول در رودخانه زاینده‌رود

http://hsr.mui.ac.ir/index.php/jhsr/article/view/44

مقدمه: رودخانه زاینده‌رود تنها رودخانه حوضه مرکزی ایران است که دارای آب شیرین دایمی می‌باشد. این رودخانه اهمیت بسیاری در تأمین آب آشامیدنی، حفظ حیات صنعتی استان و تأمین آب کشاورزی دارد. قسمت بالا دست این رودخانه (بالا دست بند چم‌آسمان) به دلیل تأمین آب شرب از اهمیت بالایی برخوردار است. بنابراین با توجه به رشد صنایع، افزایش جمعیت و عوامل متعدد تأثیرگذار بر کیفیت منابع آب، مدیریت این منبع نیاز به ابزارای قدرتمند و به روز دارد. روش‌ها: استفاده از مدل‌های کمی و کیفی مانند مدل 11 MIKE می‌تواند ابزاری کارآمد جهت مدیریت این شریان حیاتی محسوب شود. در این مطالعه به منظور شبیه‌سازی کمی و کیفی رودخانه زاینده‌رود، از حد فاصل سد تنظیمی تا پل ‌کله از نرم‌افزار 11 MIKE که یک مدل کیفیت آب با هسته هیدرودینامیک می‌باشد، استفاده شده است. بدین منظور در مدت پنج ‌ماه از چهار ایستگاه هیدرومتری نمونه‌برداری به عمل آمد و میزان اکسیژن محلول، pH، BOD یک روزه، سه روزه، پنج روزه و هفت روزه، NH4، NO3، فسفات و دما اندازه‌گیری شد. دیگر داده‌های مورد نیاز مدل شامل داده‌های مورفولوژیک و هیدرولوژی و هواشناسی از سازمان آب منطقه‌ای اصفهان و اداره هواشناسی شهرکرد تهیه و به مدل معرفی گردید. در مرحله بعد ضمن واسنجی مدل، دقت مدل مورد بررسی قرار گرفت. نتایج همبستگی پیرسون نشان داد نتایج مدل و نتایج میدانی از همبستگی مناسبی برخوردار است و ضریب همبستگی در اکثر پارامترها از 9/0 بیشتر است. در انتها وضعیت رودخانه در 25 سال آینده تحت دو سناریو مورد بررسی قرار گرفت. یافته‌ها: نتایج این پژوهش نشان می‌دهد غلظت فسفات، BOD5، COD، NH4،  NO3 در رودخانه زاینده‌رود، از سد تنظیمی تا پل کله از استاندارد مجاز آب‌های سطحی بالاتر می‌باشد. نتایج پیش‌بینی مدل جهت 25 سال آینده، گویای این مطلب است که با توجه به روند رو به رشد جمعیت و صنایع، ورود آلاینده‌ها به این شریان حیاتی افزایش می‌یابد و در نتیجه غلظت آلاینده‌ها در رودخانه زیادتر می‌شود و این افزایش نه تنها محیط زیست آبی را به خطر می‌اندازد بلکه تصفیه آب رودخانه به منظور تأمین آب آشامیدنی توسط تصفیه‌خانه بابا شیخعلی را نیز با مشکل مواجه می‌سازد. نتیجه‌گیری: نتایج مشاهدات مؤید آن است که غلظت اکسیژن محلول جهت محیط زیست آبی مناسب است اما این مقدار اکسیژن محلول به منظور مهاجرت و تخم‌ریزی ماهیان، کافی نمی‌باشد.

Title: تأثیر رژیم كم كالری غنی از حبوبات بر روی شاخص‌های تن‌سنجی در زنان با چاقی شكمی

http://hsr.mui.ac.ir/index.php/jhsr/article/view/138

مقدمه: شناسایی راه‌های جدید درمان چاقی برای سلامت بشر از اهمیت فوق‌العاده‌ای بر خوردار است.  هدف از اين مطالعه پاسخ به این پرسش بود كه آیا مكمل خوراكی ال‌-‌آرژنین و سلنیوم به تنهایی یا توأم با هم می‌توانند تأثیر رژیم كم‌ كالری غنی از حبوبات را بر روی شاخص‌های تن‌سنجی افزایش دهد؟ روش‌ها: این کارآزمایی بالینی دوسوکور کنترل‌شده با دارونما بر روی 84 خانم قبل از یائسگی و با چاقی مرکزی اجرا گردید. شرکت‌کنندگان در مطالعه پس از دو هفته اجرای رژیم آماده‌سازی ایزوکالریک به صورت تصادفی یکی از 4 مداخله‌ي 1) رژیم كنترل ، 2) ال-‌آرژنین به همراه رژیم كنترل، 3) سلنیوم به همراه  رژیم كنترل 4) ال-آرژنین، سلنیوم و رژیم كنترل را به مدت 6 هفته دريافت نمودند. شاخص‌هاي تن‌سنجي قبل و بعد از مداخله مورد سنجش قرار گرفته شد. یافته ها: رژیم كنترل تأثیر معنی‌داری در كاهش دور كمر، باسن، بازو، ران، ساق پا، سینه و ضخامت چین پوستی عضلات سه ‌سر و دو‌ سر ‌بازو، زیر كتف و فوق‌ایلیاك، نسبت دور كمر به دور باسن، مجموع ضخامت‌های چین پوستی، دانسیته‌ی بدن و درصد چربی بدن داشت. رژیم كنترل به‌ همراه ال-‌آرژینین و سلنیوم به طور معنی‌دار ضخامت چین پوستی فوق‌ایلیاك را كاهش داد ولی رژیم كنترل، ال-‌آرژینین، سلنیوم هر کدام به تنهایی یا با همدیگر تأثیر معنی‌داری بر وزن، نمایه توده بدن و غلظت ناشتای نیتریت (نیترات) نداشتند. نتیجه‌گیری: رژیم كنترل به همراه ال-‌آرژنین و سلنیوم چاقی شكمی را كاهش مي‌دهد.

Title: بررسی تاثیرآموزش با استفادهبررسی تاثیرآموزش در پیشگیری از مصرف سیگار در دانش آموزان شهر زاهدان

http://hsr.mui.ac.ir/index.php/jhsr/article/view/133

مقدمه: استعمال دخانيات يكي از بیشترین علل مرگ و مير زود هنگام است. هدف از این مطالعه بررسی تاثیرآموزش با استفاده از الگوی اعتقاد بهداشتی در پیشگیری از مصرف سیگار در دانش آموزان دوم دبیرستان شهر زاهدان بود. روش‌ها: در این پژوهش  نیمه تجربی 380 نفر از طریق نمونه‌گیری تصادفی خوشه‌ای انتخاب و به صورت تصادفی به دو گروه کنترل و آزمون تقسیم شدند. ابزار گردآوری اطلاعات پرسش‌نامه‌ای شامل سئوالات دموگرافیک، آگاهی، نگرش و سئوالاتی بر اساس سازه‌های مدل اعتقاد بهداشتی بود. روایی و پایایی آن از طریق روایی محتوایی و آزمون مجدد تعیین شد و بر اساس آن پیش آزمون از هر دو گروه به عمل آمد. مداخله آموزشی از طریق سخنرانی به همراه پمفلت بر روی گروه آزمون انجام شد. دو ماه پس از مداخله از طریق همان پرسش‌نامه پس آزمون به عمل آمد. اطلاعات بدست آمده با نرم افزار آماری 18SPSS و از طریق آزمون کای دو، ضريب همبستگي پيرسون وt-student مورد تجزیه و تحلیل قرار گرفت. یافته‌ها: قبل از مداخله، میانگین نمرات دو گروه از نظر سطح آگاهی، نگرش، منافع، موانع، حساسیت، شدت درک‌شده، راهنمای عمل و خود کار آمدی تفاوت معنی‌داری نداشتند. پس از آموزش میانگین نمرات تمام متغیرهای فوق در گروه آزمون به طور معنی‌داری افزایش پیدا کرد. ولی میانگین نمرات گروه کنترل تفاوت معنی‌داری پیدا نکرد. نتیجه‌گیری: نتایج این مطالعه حاکی از تاثیر مداخله آموزشی بر اساس سازه‌های‌ مدل اعتقاد بهداشتی در رفتار پیشگیری‌کننده از مصرف سیگار در بین دانش آموزان پایه دوم دبیرستان شهر زاهدان است. واژه‌های ‌کلیدی‌: آگاهی، نگرش، مصرف سیگار، مدل اعتقاد بهداشتی، آموزش بهداشت

Title: مقايسه کارايي امواج مايکروويو با کوره‌های متداول الکتريکی در کاهش حجم و تثبيت نهايي لجن‌های خام اوليه و ثانويه توليدی در تصفيه‌خانه فاضلاب جنوب اصفهان

http://hsr.mui.ac.ir/index.php/jhsr/article/view/43

مقدمه: جامدات بيولوژيکی توليدی حين عمليات تصفيه فاضلاب‌ها، لجن ناميده می‌شوند. در واقع بخش اعظم آلاينده‌هايي که مسؤول ماهيت بد، نفرت‌انگيز و زيان‌آور در فاضلاب هستند، در لجن تغليظ شدند که حاوی انواع ميکروارگانيسم‌های بيماري‌زا، مواد آلی و معدنی می‌باشند و می‌توانند برای انسان و محيط زيست مضر و خطرناک باشند. بنابراین قبل از اين که در محيط رها شوند، بايد کاملاً تصفيه و پردازش شوند و با توجه به مقررات زيست محيطی دفع گردند. يکی از روش‌هايي که برای تصفيه لجن مورد استفاده قرار می‌گیرد، تصفيه حرارتی است. در اين ميان به نظر می‌رسد که امواج مايکروويو به عنوان يکی از اين روش‌ها می‌تواند نقش مهمی در تصفيه حرارتی داشته باشد. روش‌ها: در اين مطالعه، كارايي امواج مذکور جهت کاهش حجم و تثبيت نهايي لجن‌‌‌‌‌های اوليه و ثانويه حاصل از تصفيه‌خانه فاضلاب جنوب اصفهان در مقايسه با کوره متداول الکتريکی آزمايشگاهی مورد بررسي علمي قرار گرفت. در اين مطالعه، آزمايش‌ها به روش ناپيوسته و با استفاده از نمونه‌های لجن اوليه و ثانويه حاصل از تصفيه‌خانه فاضلاب جنوب اصفهان و طی دو مرحله (به تنهايي و با افزودن ماده جاذب) انجام شد و تأثير متغيرهايي مانند مدت زمان اشعه‌دهی در فاصله 1 تا 10 دقيقه برای اجاق مايکروويو، 240 و 300 دقيقه برای حرارت‌دهی کوره الکتريکی، تأثیر ميزان ماده جاذب بين 5 تا 30 درصد وزنی بر مقدار دمای ايجاد شده در توده لجن، درصد کاهش وزن، درصد کاهش جامدات کل و فرار، کاهش باکتري‌های کليفرم و شمارش بشقابی باکتري‌های هتروتروفيک آزمايش گرديد. یافته‌ها: با افزايش زمان اشعه‌دهی، کارايي اجاق مايکروويو در کاهش وزن لجن و حذف درصد رطوبت محتوی نمونه‌ها افزايش يافت و در نمونه‌هايي که درصدهای مختلفی ماده جاذب اشعه (مانند زغال چوب) به آن‌ها افزوده شده بود، دمای توده نمونه‌ها تا حدود 600 درجه سانتيگراد افزايش يافت، در حالي‌ که در دمای نمونه‌های مشابه مورد عمل در کوره الکتريکی، افزايش دمايي قابل توجهی مشاهده نگرديد. همچنين در نمونه‌های بدون ماده جاذب، اجاق مايکروويو قادر به کاهش تا 5/4 و log 5 به ترتيب برای باکتري‌های کليفرم و شمارش بشقابی هتروتروفيک بود. در نمونه‌های حاوی ماده جاذب، تا 100 درصد اين ميکروارگانيسم‌ها حذف گرديدند. نتیجه‌گیری: اطلاعات حاصل از اين تحقيق در مجموع مشخص مي‏سازند که در صورتي كه نمونه‌های لجن به تنهايي تحت اشعه مايکروويو در زمان‌های مختلف قرار گيرند، فقط خشک می‌گردند و رطوبت خود را از دست مي‌دهند؛ اما اگر به آن‌ها درصدهايي از مواد جاذب اشعه مانند زغال چوب افزوده شود، علاوه بر خشک شدن، به دليل افزايش بيش از حد دما در توده نمونه‌ها، احتمال رخ دادن فرايند پيروليز در آن‌ها وجود دارد و نمونه‌های لجن ضمن هضم شدن، تثبيت می‌گردند.

Title: بررسی کارایی تجزیه هیدروکربور نفتی زایلن با استفاده از تابش خورشیدی به روش UV/TiO2 در پساب صنعتی سنتتیک

http://hsr.mui.ac.ir/index.php/jhsr/article/view/54

مقدمه: ترکیبات بنزن، تولوئن، اتیل بنزن و زایلن (BTEX یا Benzene, Toluene, Ethyl benzene and Xylene) در محیط‌های آبی از لحاظ بهداشتی و زیست محیطی بسیار خطرناک بوده، دارای سمیت بالا می‌باشند. هدف این مطالعه، بررسی کارایی تجزیه فتوکاتالیستی هیدروکربن نفتی زایلن در پساب صنعتی سنتتیک به روش UV/TiO2 با استفاده از تابش خورشیدی بود. روش‌ها: این مطالعه از نوع تجربی مداخله‌ای بوده، که در مقیاس آزمایشگاهی انجام شد. رآکتور مورد استفاده دارای پنج مدول شیشه‌ای متصل به هم با قطر cm 3 و یک موتور گرداننده جهت حداکثر دریافت اشعه UV خورشید بود. برای تعیین عوامل مؤثر بر راندمان حذف- پاسخ، از نسبت سیگنال به نویز استفاده شد که با استفاده از فرمول  به دست می‌آید. یافته‌ها: طبق نتایج به دست آمده، غلظت ورودی زایلن بیشترین تأثیر را بر راندمان حذف داشت و فاکتورهای مؤثر بعدی به ترتیب pH با مقدار 7، زمان با محدوده 60 تا 180 دقیقه و غلظت TiO2 با مقدار mol/L 01/0 بودند. شدت اشعه آفتاب در محدوده w/cm2 45 تا 60 و درجه حرارت در محدوده 40 تا 60 درجه سانتی‌گراد بود. نتیجه‌گیری: روش تاگوچی در تجزیه و حذف هیدروکربن نفتی زایلن، ارتباط معنی‌دار آماری بین راندمان حذف با فاکتورهای مورد آزمایش نشان داد و در همه موارد 1/0 < P به دست آمد. غلظت زایلن بیشترین تأثیر را در کارایی تجزیه داشته، با افزایش آن کارایی حذف افزایش یافت. به طوری‌ که در شرایط بهینه 33/93 درصد زایلن حذف گردید.

Title: ارتباط بین حمایت اجتماعی و سلامت روان در دانشجویان دانشگاه تهران

http://hsr.mui.ac.ir/index.php/jhsr/article/view/29

مقدمه: دانشگاه‌ها، سازمان‌هايي هستند كه هر ساله مقدار قابل توجهي جمعيت جوان را جذب مي‌كنند و طي زمان معيني توانمندي‌هاي علمي و عملي اين اشخاص را پرورش مي‌دهند و در پایان نيروهاي آموزش ديده و متخصص را در اختيار جامعه مي‌گذارند هدف از این مطالعه بررسی رابطه بين حمايت اجتماعي، سلامت رواني و متغيرهاي جمعيت شناختي در دانشجويان ورودي جديد دانشگاه تهران در سال تحصيلي1385- 1384- بود. روش‌ها: اين مطالعه از نوع توصيفي به روش نمونه‌گيري سرشماري روی 2916 دانشجو ورودي جديد سال تحصيلي 1385-1384 (1176مرد و1737 زن) در دانشگاه تهران انجام شده است‌ دانشجويان پذيرفته شده در محدوده زماني ثبت‌نام به غرفه مركز مشاوره دانشجويي مراجعه و با راهنمايي كارشناسان، از سه پرسش‌نامة مشخصات جمعيت شناختي، سلامت عمومی12 سؤالي ((GHQ-12 و مقياس حمايت اجتماعی استفاده کردند. تحليل آماري داده‌ها با آزمون‌هاي t، ANOVA و Regression انجام شد. یافته‌ها: ميانگين سني اين دانشجويان 61/1 19، میانگین نمره سلامت عمومی‏69/4 53/9 و حمايت اجتماعي 21/2 15/10 بود. نتیجه آزمون نشان داد که با افزايش ميزان حمايت اجتماعي نمره سلامت عمومي كاهش يافته (P<0.001, b=-0.57, ß=-0.27, t=-14.66) یعني وضعيت سلامت عمومي بهبود مي‌يابد. نتیجه‌گیری: حمایت اجتماعی با سلامت روان رابطه معنی‌دارو مستقیم دارد. تغيير سياست‌گذاري‌ها با هدف ارتقاء كيفيت و كميت شبكه‌هاي حمايتي، به ويژه براي دانشجويان آسيب‌پذير، مي‌تواند باعث تقويت اثرات محافظتي حمايت‌هاي اجتماعي شود..

Title: بررسي افسردگي در بيماران ديابتي نوع 2 اصفهان

http://hsr.mui.ac.ir/index.php/jhsr/article/view/30

مقدمه: دیابت یکی از بیماری‌های شایع در جهان امروز است که تعداد مبتلایان به آن در دنیا به 177 میلیون نفر می‌رسد در افراد مبتلا به دیابت شانس ابتلا به افسردگی نسبت به غیر مبتلایان به شکل قابل توجه بیشتر می‌باشد، خصوصیات زمینه‌ای فرد نیز در بروز اختلالات روانی می‌تواند مؤثر باشد. این مطالعه با هدف ارزیابی شدت افسردگی در بیماران دیابتی با توجه به خصوصیات زمینه‌ای انجام گردید.  روش‌ها: در این مطالعه مقطعی 383 نفر از بیماران دیابتی مراجعه کننده به انجمن خیریۀ اصفهان در سال 1386 به روش نمونه‌گیری سیستماتیک انتخاب شدند و از نظر ابتلا به افسردگی توسط پرسش‌نامه دو بخشی که شامل مشخصات زمینه‌ای و 21 سؤال تست استاندارد شدۀ بک (Beck) بود، مورد ارزیابی قرار گرفتند.افرادی که نمره آنان بین 9-0 بوده، بدون افسردگی، نمرۀ 16– 10 افسردگی خفیف، نمرۀ 29-17 افسردگی متوسط، نمرۀ 63-30 افسردگی شدید درنظر گرفته شد پس از تکمیل پرسش‌نامه، داده‌ها با استفاده از نرم افزار SPSS و آزمون‌های آماری مورد تجزیه و تحلیل قرار گرفت. یافته‌ها: 107 نفر (28 درصد) بیماران دیابتی دارای خلق طبیعی،82 نفر (4/21 درصد) بیماران دارای افسردگی خفیف، 136 نفر (5/35) درصد دارای افسردگی متوسط و 58 نفر 1(/15 درصد) بیماران افسردگی شدید داشته‌اند. شدت افسردگی در زنان (5/56 درصد) به شکل معنی‌داری نسبت به مردان (8/36 درصد) بیشتر بود (P<0.01). ارتباط معنی‌دار معکوس بین شدت افسردگی و سن مشاهده شد (P=-0.02). نتیجه‌گیری: بالا بودن میزان افسردگی در بیماران دیابتیک بود، انجام مشاوره روانپزشکی جهت بررسی افسردگی در افراد دیابتی توصیه می‌شود.

Title: استفاده از رویکرد پویایی سیستم جهت مدل‌سازی اوتریفیکاسیون در دریاچه‌های مصنوعی

http://hsr.mui.ac.ir/index.php/jhsr/article/view/53

مقدمه: امروزه با ورود انواع پساب‌ها و رواناب‌ها به منابع آب، فرایند اوتریفیکاسیون یا تغذیه‌گرایی به شدت تسریع شده است. چنین است كه نیاز به مدیریت دریاچه‌های طبیعی و مصنوعی، به منظور پیش‌گیری یا به تعویق انداختن این مشكل، به وجود آمده است. تغذیه‌گرایی یك فرایند پیچیده است. یكی از ابزارهایی كه در این زمینه می‌تواند ما را یاری كند، مدل‌سازی فرایند تغذیه‌گرایی می‌باشد. امروزه مدل‌های زیادی وجود دارد كه این كار را انجام می‌دهد. نیاز به طیف وسیعی از داده‌ها، یادگیری مشكل و نیاز به زمان طولانی از مشكلات این مدل‌ها می‌باشد. امروزه جهت مدیریت تغذیه‌گرایی اغلب از ابزارهای كامپیوتری، به ‌ویژه مدل‌سازی ریاضی بهره گرفته می‌شود.  روش‌ها: در این مقاله یك مدل فرایندی ساخته شد. نخست سیستم تغذیه‌گرایی، مشخصات و پارامترهای آن تعیین و روی صفحه VenSim ایجاد شد. سپس روابط ریاضی هر فرایند تعیین و وارد كامپیوتر شد. در نهایت مدل با استفاده از داده‌های یك مخزن، كالیبره و تأیید شد. این مدل متغیرهای اصلی تغذیه‌گرایی را مدل می‌كند. این متغیرها شامل فسفر، نیتروژن، فیتوپلانكتون و زئوپلانكتون است. یافته‌ها: با بررسی نتایج حاصل از مدل، یك شكوفایی جلبكی مشاهده شد. در كالیبراسیون مدل، ثابت نیم اشباع نیتروژن برابر 10 میلی‌گرم بر لیتر بر حسب نیتروژن، ثابت نیم اشباع فسفر برابر 5 میلی‌گرم بر لیتر بر حسب فسفر به دست آمد. نتیجه‌گیری: یافته‌های مدل نشان داد كه فسفر یك عامل محدود كننده در مخزن است. نتایج حاصله بیان‌گر آن است كه در مقایسه با مدل‌های دیگر، استفاده از این مدل برای كاربر آسان‌تر بوده، با داشتن كمترین داده‌ها، نتایج مطلوب حاصل می‌گردد.

Title: بررسي ارتباط بین سطح سرمی هموسیستئین و لیپوپروتئین (a) با ابتلا به بيماري‌هاي قلبي

http://hsr.mui.ac.ir/index.php/jhsr/article/view/161

مقدمه: همگام با پيشرفت تکنولوژي‌هاي پيشرفته پزشکي و روش‌هاي تشخيصي درماني بيماران قلبي، عوامل خطر جديدي در اتيولوژي بيماري‌هاي قلبي- عروقي مطرح مي‌شود. هموسيستئين، ليپوپروتئين (a)، قيبرينوژن و فاکتور هفت از جمله عوامل خطر جديد بيماري‌هاي قلبي- عروقي هستند که توجه پژوهشگران را به خود جلب نمودند. هدف از اين مطالعه موردی- شاهدي بررسي ميزان هموسيستئين در بيماران قلبي بستري در بيمارستان شهيد مصطفي خميني در شهر ايلام و مقایسه آن با افراد گروه شاهد است.  روش‌ها: در اين مطالعه موردی- شاهدي، افراد گروه مورد را بيماران قلبي بستري در بخش قلب (40 n =) بیمارستان شهید مصطفی خمینی ایلام و افراد گروه شاهد (60 n =) را کسانی تشکیل دادند که بدون هيچ‌گونه سابقه بيماري قلبي در این بیمارستان برای منظور‌های دیگری غیر از بیماری‌های قلبی بستری شدند. پس از کسب رضايت کتبي از کليه نمونه‌ها 8 سي‌سي نمونه خون ناشتا جهت بررسي هموسيستئين، قند خون ناشتا (FBS) کلسترول، تري گليسريد، LDL و HDL گرفته شد. داده‌هاي دموگرافيک و باليني با استفاده از يک پرسش‌نامه استاندارد جمع‌آوری گرديد. يافته‌ها: بيش از نيمي از افراد گروه مورد (5/52 درصد) زن و بيش از يک سوم افراد گروه شاهد (6/36 درصد) مرد بودند. ميانگين سني و انحراف معيار افراد گروه مورد و شاهد به ترتيب 27/10 ± 48/49 و 03/13 ± 25/42 سال بود (05/0 P >). ميانگين سطح سرمي هموسيتئين در افراد گروه مورد و شاهد به ترتيب 02/3 ± 51/13 و 10/3 ± 42/13 بود. غلظت لیپوپروتئین (a) در گروه مورد و شاهد به ترتیب 66/51 ± 22/35 و 96/17 ± 96/13 بود. در هر دو گروه با افزايش سن غلظت هموسيستئين افزايش قابل توجهي داشت که اين رابطه فقط در گروه مورد معني‌دار بود (006/0 = (P. غلظت قند خون ناشتا، تري گليسريد، LDL و کلسترول در گروه مورد به ميزان قابل ملاحظه‌اي بالاتر از گروه شاهد بود.  نتيجه‌گيري: نتايج اين مطالعه نشان می‌دهند که بين افزایش سطح هموسيستئين و افزایش سن ارتباط معنی‌داری وجود دارد و از آن جا که سن بالا، خود برای بيماري‌هاي قلبي فاکتور مساعد کننده‌ای است بنابراین این ریسک فاکتور نقش مؤثری در افزایش احتمال ابتلای به بیماری‌های قلبی مورد تأييد قرار مي‌دهد هر چند که ارتباط این ریسک فاکتور با فاکتور‌های خطر دیگر در گروه‌های مورد و شاهد از نظر آماری معنی‌دار نمی‌باشد؛ ارتباط بین هموسیستئین و HDL از نظر آماری معنی‌دار است، ولی نتایج این مطالعه حاکی از این است که نقش هموسیستئین به عنوان یک ریسک فاکتور جدید به اندازه نقش TG، کلسترول و LDL در شیوع و شدت بیماری‌های قلبی محرز نیست و از این رو برای حصول اطمینان از چنین ارتباطی انجام مطالعات بيشتر با حجم نمونه بالاتر توصيه مي‌گردد.

Title: بررسی ارتباط بین میزان دریافت تنقلات و وزن در کودکان 7-6 ساله شهرستان شاهین شهر و میمه در سال 1388

http://hsr.mui.ac.ir/index.php/jhsr/article/view/149

مقدمه: بسیاری از عادات و الگوهای غذایی در دوران کودکی شکل می‌گیرند و تا پایان زندگی فرد باقی می‌مانند. تغذیه صحیح در این دوران، سبب ارتقای رشد و تکامل کودک می‌شود، به علاوه از خطر ابتلا به بیماری‌های مزمن در دوران بزرگسالی می‌کاهد (1). کودکان سنین دبستان، گروه خاصی را از نظر آسیب پذیری اجتماعی، بهداشتی و سلامت تغذیه تشکیل می‌دهند. چرا که کودکان 7 ساله، اولین گروه سنی هستند که وارد محیط جدید مدرسه می‌شوند و اغلب این موضوع باعث پدید آمدن تغییراتی در برخی عادات و شیوه زندگی، به ویژه عادات غذایی آنان می‌گردد. همچنین میزان رشد و سلامت، ارتباط تنگاتنگی با کیفیت و کمیت غذای مصرفی دارد و اطلاعات مربوط به وضعیت سلامت و تعیین وضعیت تغذیه‌ای این گروه از نظر شیوع کم وزنی، چاقی و عادات غذایی از مسایل مهم بهداشتی است (3، 2). مطالعات زیادی در نقاط مختلف کشور بر روی کودکان انجام شده است که به شیوع کم وزنی و اضافه وزن در این گروه سنی اشاره دارد (8-4). روش‌ها:این مطالعه از نوع مقطعی و به صورت توصیفی- تحلیلی بود. افراد مورد مطالعه کودکان 7-6 ساله شهرستان شاهین شهر و میمه بودند.کودکان با وزن نرمال به روش نمونه‌گیری تصادفی ساده از مدارس ابتدایی شهرستان و کودکان با وزن زیر صدک 5ام و بالای صدک 95ام استاندارد NCHS، از طرح کشوری غربالگری سنجش در بدو ورود به مقطع ابتدایی انتخاب شدند. جمع‌آوری اطلاعات کلی کودک (شامل نام، تاریخ تولد و ...) با استفاده از پرسش‌نامه و بررسی وضعیت مصرف مواد غذایی با استفاده از پرسش‌نامه تکرر مواد غذایی انجام گردید و روایی و پایایی  FFQمورد استفاده، مورد تأیید قرار گرفته بود (2). وزن کودک بدون کفش با حداقل لباس با استفاده از ترازوی استاندارد با دقت 1/0 کیلوگرم، قد با قدسنج با دقت 1 سانتی‌متر در حالی که کتف‌ها در حالت عادی و نگاه شخص به روبه‌رو باشد، اندازه‌گیری و سن کودک بر اساس سال و ماه مشخص شد. ضمن این‌که نمودارهای استاندارد  NCHSبرای وزن و قد در شناسنامه سلامت هر دانش آموز رسم شده بود. یافته‌ها: میانگین و انحراف معیار مصرف تنقلات (چیپس، پفک وکرانچی، انواع شکلات،آبنبات وکاکائو، انواع بیسکویت، انواع کیک، نوشابه وآبمیوه‌های صنعتی) در کودکان با وزن زیر صدک 5ام استاندارد NCHS 58/21 ± 43/38، در کودکان با وزن نرمال 90/21 ± 65/34 و در بین کودکان با وزن بالای صدک 95ام استاندارد NCHS ، 18/38 ± 29/40 بار در هفته به دست آمد و نشان دهنده این بود که میزان مصرف تنقلات به ترتیب در کودکان با وزن بالای صدک 95ام استاندارد NCHS و کودکان با وزن زیر صدک 5ام استاندارد NCHS، بالاتر از کودکان با وزن نرمال بود. ولی ارتباط معنی‌داری بین مصرف تنقلات و وزن کودکان مشاهده نشد. در کودکان با وزن زیر صدک 5ام و کودکان بالای صدک 95ام استاندارد NCHS، ارتباط معنی‌داری بین میزان تحصیلات مادر و پدر و میزان دریافت تنقلات مشاهده نشد، ولی در کودکان با وزن نرمال استاندارد، ارتباط معنی‌داری (007/0 = P) بین میزان تحصیلات پدر و میزان دریافت تنقلات مشاهده شد. میانگین و انحراف معیار مدت زمان تغذیه انحصاری با شیر مادر در کودکان با وزن زیر صدک 5ام 12/5 ± 05/7، درکودکان با وزن نرمال 80/2 ± 98/5 و در کودکان با وزن بالای صدک 95ام 70/2 ± 89/5 ماه می‌باشد. بیشترین تنقلات مصرفی در این کودکان کیک، بیسکویت و شربت بود. میانگین تعداد وعده‌‌های غذایی در کودکان کم وزن، نرمال و چاق، به ترتیب 3/1 ± 19/4، 09/1 ± 96/3 و 09/1 ± 91/4 به دست آمد و ارتباط بین صدک وزن کودک با تعداد وعده‌های غذایی، با آزمون همبستگی پيرسون 002/0 ± 023/0 به دست آمد که بیانگر ارتباط معنی‌دار بین صدک وزن کودک با تعداد وعده‌های غذایی است. از کل نمونه، 2/71 درصد به طور منظم مکمل آهن و مولتی ویتامین را قبل از 2 سالگی دریافت می‌کردند و 8/28 درصد مکمل آهن و مولتی ویتامین را به طور منظم دریافت نمی‌کردند. ارتباط بين وزن و مصرف منظم مکمل آهن و مولتی ویتامین قبل از 2 سالگی با استفاده از ضريب همبستگی پيرسون سنجيده شد. نتايج اين طرح ارتباط معنی‌داری را بین مصرف منظم مکمل‌های آهن و مولتی ویتامین با وزن کودک نشان نداد، البته اين امر ممکن است به دلیل کوچک بودن حجم نمونه باشد. از کل نمونه‌ها 2/76 درصد صبحانه مصرف می‌کردند. درصد مصرف صبحانه در کودکان کم وزن، نرمال و چاق به ترتیب 3/80 درصد، 5/84 درصد، 5/62 درصد بود. ارتباط معنی‌دار بین صدک وزن کودک و مصرف صبحانه مشاهده نشد. نتيجه‌گيري: مصرف بی‌رویه تنقلات از یک سو از طریق کاهش اشتها، فرصت تغذیه با غذاهای مغذی سفره خانوار را ازکودک می‌گیرد. از سوی دیگر به علت این‌که تنقلات حاوی قند، نمک و چربی زیاد و کالری بالا هستند، زمینه را برای چاقی کودکان فراهم می‌کنند. نتایج مطالعه کنونی در کودکان با وزن نرمال استاندارد، ارتباط معنی‌داری (007/0 = P) را بین میزان تحصیلات پدر و میزان دریافت تنقلات نشان داد. میزان تحصیلات و آگاهی تغذیه‌ای والدین از طریق تهیه میان وعده‌های مناسب به جای تنقلات نا سالم برای کودکان می‌تواند در حفظ وزن نرمال آنان مؤثر باشد. ارتباط معنی‌داری بین مصرف تنقلات و وزن کودکان در سه گروه زیر صدک 5ام، نرمال و بالای صدک 95ام استاندارد NCHS مشاهده نشد.

Title: بررسی ارتباط شاخص‌های التهابی (CRP) و شاخص‌های تغذیه‌ای (آلبومین پلاسم و دریافت مواد مغذی) در بیماران همودیالیزی

http://hsr.mui.ac.ir/index.php/jhsr/article/view/169

مقدمه: سوء تغذیه پروتئین انرژی و التهاب در بین بیماران دیالیزی شایع می‌باشد. سوء تغذیه پیامدهای بسیاری از جمله افزایش مرگ و میر و دیر التیام یافتن زخم‌ها، افزایش حساسیت به عفونت، خستگی و بی‌حالی به دنبال دارد. سوء تغذیه پروتئین انرژی و التهاب در بیماران همودیالیزی اغلب منجر به سندرم کمپلکس سوء تغذیه التهاب یا آترواسکلروز– سوء تغذیه و التهاب می‌گردد، که با بیماری آترواسکلروتیک قلبی عروقی ارتباط دارد. این سندرم در بیمارانی که به مدت طولانی تحت دیالیز هستند، ایجاد می‌گردد و عامل مهم مرگ و میر در این بیماران محسوب می‌شود. با توجه به این که مطالعات مختلف نتایج متفاوتی را در مورد ارتباط التهاب و سوء تغذیه نشان می‌دهد، بر آن شدیم تا ارتباط شاخص‌های التهابی و دریافت مواد مغذی را در بیماران همودیالیزی بررسی کنیم. روش‌ها: این پژوهش به روش مقطعی بر روی 35 نفر از بیماران همودیالیز (5/58 درصد مرد و 1/48 درصد زن با میانگین سنی 55/51 سال) انجام شد، که حداقل به مدت 6 ماه تحت دیالیز بودند. اطلاعات به وسیله یک پرسش‌نامه حاوی مشخصات فردی، پزشکی و آزمایشات کلینیکی و یک پرسش‌نامه بسامد خوراکی (FFQ یا Food Frequency Questionnaire) جمع‌آوری گردید. مشخصات آنتروپومتریک شامل قد و وزن با استفاده از ترازوی Seca و قد‌سنج اندازه‌گیری گردید و نمونه خون از بیماران گرفته شد و جهت انجام آزمایشات به آزمایشگاه ارسال گردید. پرسش‌نامه بسامد خوراکی شامل 194 قلم غذایی از کلیه افراد تکمیل گردید و نتایج به دست آمده با نرم‌افزار N3 و SPSS تجزیه و تحلیل گردید. یافته‌ها: پس از پایان مطالعه ارتباط بین دریافت سلینوم و ویتامین B1 با CRP معنی‌دار بود (001/0 = P). در مورد بقیه مواد مغذی مورد بررسی ارتباط معنی‌داری مشاهده نشد. در ضمن دریافت پروتئین، کالری، فیبر، ویتامین‌ها و املاح به طور معنی‌داری پایین‌تر از DRI بود. نتیجه‌گیری: با توجه به این که دریافت کالری، پروتئین و تعدادی از ویتامین و املاح در این افراد به طور معنی‌داری کمتر از DRI بود و همچنین ارتباط بعضی مواد مغذی با CRP معنی‌دار بود، انتظار می‌رود که این افراد دچار سیکل سوء تغذیه– التهاب شده باشند، یا به زودی وارد این سیکل گردند، که این اهمیت مراقبت‌های تغذیه‌ای را در بهبودی و پیش‌گیری از مرگ و میر در این افراد را نشان می‌دهد.

Title: بررسی میزان تراکم آئروسل‌ها و بیوآئروسل‌ها در کارگاه‌های قالیبافی خانگی روستایی اصفهان

http://hsr.mui.ac.ir/index.php/jhsr/article/view/101

مقدمه: بیشتر کارگاه‌های قالیبافی روستایی بخشی از منزل مسکونی بوده و شرایط بهداشتی موجود در کارگاه نه تنها قالیبافان بلکه سایر افراد خانواده را تحت تأثیر قرار می‌دهد. این مطالعه با هدف تعیین میزان تراکم آئروسل و بیوآئروسل در کارگاه‌های قالیبافی خانگی روستایی اصفهان انجام پذیرفت. روش‌ها: پژوهش حاضر یک مطالعه توصیفی– تحلیلی بود که روی 92 کارگاه قالیبافی خانگی روستایی اصفهان انجام پذیرفت. برای نمونه‌برداری آئروسل‌ها از پمپ نمونه‌بردار فردی و نمونه‌بردار IOM و برای نمونه‌برداری بیوآئروسل‌ها از دستگاه نمونه‌بردار باکتریایی استفاده گردید. نمونه‌ها پس از جمع‌آوری به آزمایشگاه آنالیز ارسال گردیدند. داده‌ها با نرم‌افزار SPSS مورد تجزیه و تحلیل قرار گرفتند. یافته‌ها: میانگین میزان تراکم آئروسل در کارگاه‌ها  mg/m36/1± 4/ 2و میانگین میزان تراکم بیوآئروسل‌هاColony/m3 332± 430 به دست آمد. بررسی ارتباط بین میزان تراکم آئروسل و نشان دهنده ارتباط مستقیم بین این دو متغیر بود، اما این ارتباط از نظر آماری معنی‌دار نبود. میانگین تراکم بیوآئروسل‌ها با وجود دام و طیور در حیاط ارتباط معنی‌دار آماری نشان داد (031/0 = P). نتیجه‌گیری: میانگین گرد و غبار در کارگاه‌های قالیبافی از حد مجاز گرد و غبار کلی برای محیط‌های شغلی (    = TLVmg/m3) ) 10TLV = (بیشتر نبود، اما با توجه به این‌که محیط مورد بررسی مسکونی- شغلی بود و نیز با توجه به وجود انواع بیوآئروسل‌های پاتوژن یا پاتوژن بالقوه، چنین محیط‌های کاری یک محیط مخاطره آمیز برای شاغلین و نیز ساکنین می‌باشند. واژه‌های ‌کلیدی‌: آئروسل، بیوآئروسل، کارگاه قالیبافی روستایی خانگی

Title: مقایسه روش‌های برآورد میزان نیاز آبی صنایع بر اساس استفاده از پارامترهای تعداد واحد صنعتی، تعداد پرسنل شاغل، مساحت کل و مساحت زیربنا

http://hsr.mui.ac.ir/index.php/jhsr/article/view/67

مقدمه: کشور ایران یک کشور خشک می‌باشد که از نظر منابع آب دچار محدودیت‌های زیادی است. این مسأله در مناطق خشک و نیمه‌خشک کشور شدیدتر است. بخش صنعت یکی از مصرف کنندگان اصلی منابع آب کشور است و تعیین صحیح آب مورد نیاز صنایع از مهم‌ترین اولویت‌هایی است که باید مورد توجه قرار گیرد، تا علاوه بر صرفه جویی در مصرف آب، نیاز آبی صنایع نیز تأمین گردد. هدف از این مطالعه، انتخاب بهترین و معتبرترین روش برآورد نیاز آبی شهرک‌های صنعتی بر اساس آزمون‌های آماری معتبر بود. روش‌ها: با استفاده از آمار کنتور خوانی 1256 واحد صنعتی در 5 شهرک صنعتی واقع در استان اصفهان، اطلاعات میزان مصرف آب آن‌ها جمع‌آوری گردید. آمار میزان مصرف آب صنایع، با پارامترهای تعداد واحد صنعتی، تعداد پرسنل، مساحت کل کارخانه‌ها و مساحت زیربنای کارخانه‌ها مورد بررسی قرار گرفت. هر یک از چهار روش مذکور، برآورد مصرف آب متفاوتی را ارایه داد. یافته‌ها: با استفاده از نتایج آزمون‌های آماری، مشخص گردید که مساحت کل کارخانه و تعداد پرسنل، تحت تأثیر عوامل زیادی قرار دارند و ممکن است در ارتباط با نیاز صنایع نباشند. مساحت زیربنا تنها پارامتری است که میزان آن در ارتباط مستقیم با نیاز صنایع بوده، با میزان آب مورد نیاز یک صنعت متناسب می‌باشد. نتیجه‌گیری: برای برآورد نیاز آبی صنایع، استفاده از مساحت زیربنا نسبت به سایر گزینه‌ها، مناسب‌تر است و احتمال می‌رود که نتایج برآورد نیاز آبی با این روش به واقعیت نزدیک‌تر باشد.

Title: تأثیر آموزش براساس مدل اعتقاد بهداشتی بر عملکرد زنان در مراکز بهداشتی- درمانی شهرستان کوهدشت در مورد تست پاپ اسمیر در سال 1389

http://hsr.mui.ac.ir/index.php/jhsr/article/view/88

مقدمه: سرطان دهانه رحم دومین سرطان شایع بعد از سرطان پستان در دنیا می‌باشد و 18 درصد کل مرگ و میر در زنان به علت سرطان دهانه رحم می‌باشد. تست پاپ اسمیر یک روش غربالگری مؤثر جهت تشخیص این بیماری است. این مطالعه با هدف تعیین تأثیر آموزش بر عملکرد زنان بر اساس مدل اعتقادبهداشتی در زمینه انجام تست پاپ اسمیر انجام گرفت. روش‌ها: این مطالعه به صورت نیمه تجربی و بر روی 70 نفر به صورت تصادفی در دو گروه مورد و شاهد از زنان تحت پوشش مراکز بهداشتی درمانی کوهدشت انجام گرفت. ابزار گردآوری پرسش‌نامه‌ای مشتمل بر 4 قسمت بود. پس از جمع‌آوری اطلاعات کلاس‌های آموزشی برای گروه مورد در 2 جلسه و هر کدام در 1 ساعت انجام گرفت و پس از 1 ماه اطلاعات جمع‌آوری گردید. و با آزمون‌های آماری تجزیه و تحلیل شد. یافته‌ها‌: میانگین آگاهی بعد از مداخله در دو گروه تفاوت معنی‌داری دارد. میانگین اجزای مدل (حساسیت و شدت درک شده، منافع و موانع درک شده) تفاوت معنی‌داری در دو گروه قبل از مداخله نداشته‌اند پس از مداخله تفاوت میان دو گروه معنی‌دار شده است. قبل از مداخله نیز 51 درصد افراد کارکنان مرکز را به عنوان راهنمای به عمل انتخاب کرده‌اند که بعد از مداخله به 100 درصد رسیده است. در ارتباط با عملکرد نیز در گروه مورد و شاهد قبل از مداخله هیچ یک از نمونه‌ها تست پاپ اسمیر انجام نداده بودند اما پس از مداخله در گروه مورد 34 نفر(4/97 درصد) و در گروه شاهد تنها 1 نفر(86/2 درصد) تست مذکور را انجام داده بودند. نتیجه‌گیری: طراحی و اجرای برنامه آموزشی مبتنی بر الگوی اعتقاد بهداشتی می‌تواند سبب افزایش عملکرد زنان درخصوص انجام تست پاپ اسمیر گردد.
